# ANCA-associated systemic vasculitis initially mimicking peripheral neuropathy in an elderly woman

**DOI:** 10.12669/pjms.40.5.9070

**Published:** 2024

**Authors:** Xiaojie He, Yaqing Wang, Xiaodong Li

**Affiliations:** 1 Xiaojie He, Graduate School of Hebei Medical University, Shijiazhuang 050017, Hebei, China; 2 Yaqing Wang, Graduate School of Chengde Medical University, Chengde, 06700, Hebei, China; 3 Xiaodong Li, Department of Nephrology, Baoding No 1 Central Hospital of Hebei Medical University, Baoding, Hebei, China

**Keywords:** Anti-neutrophil cytoplasmic antibodies, Acute kidney injury, Systemic vasculitis, Peripheral neuropathy

## Abstract

Anti-neutrophil cytoplasmic antibodies (ANCA)-associated systemic vasculitis (AASV) is a rare systemic immunological condition that predominantly impacts small arteries, veins, and capillaries, often leading to kidney damage and pulmonary injury. It is important to note that individuals primarily presenting with peripheral neuropathy (PN) are uncommon in AASV, which can result in significant misdiagnosis or undiagnosed cases. The severity and location of PN can vary among patients. In this article, we present a case of an AASV patient initially showing signs of PN. This case highlights the significance of considering AASV as a potential cause of unexplained neurological symptoms. Timely identification and proper treatment are essential for improving the survival rate and functional prognosis of AASV patients.

## INTRODUCTION

Anti neutrophil cytoplasmic antibodies (ANCA)-associated systemic vasculitis (AASV) is an unusual systemic immunological disease that primarily affects small arteries, veins, and capillaries, which typically involves kidney damage and pulmonary injury. However, it can also affect skin, joints, muscles, gastrointestinal tract, and nervous system. It is worth noting that patients primarily characterized by peripheral neuropathy (PN) are rare in AASV, leading to severe misdiagnoses or missed diagnoses.

According to current studies, the incidence of PN in AASV ranges from 20% to 50%.^1^ Common symptoms of PN encompass sensory abnormalities, including but not limited to numbness, tingling sensations, and pain. Additionally, it is characterized by muscle weakness, muscle atrophy, and muscle fatigue. The severity and neural location of PN may vary among patients. Epidemiologically, men have a slight advantage over women. AASV incidence increases with age and peaks between ages 60 and 70.^2^ Herein, we report a case of an AASV patient initially presenting with PN. The objective of this case was to enhance the awareness of the diagnosis for AASV, so as to prevent any delay in its treatment.

## CASE PRESENTATION

**Fig.1:**
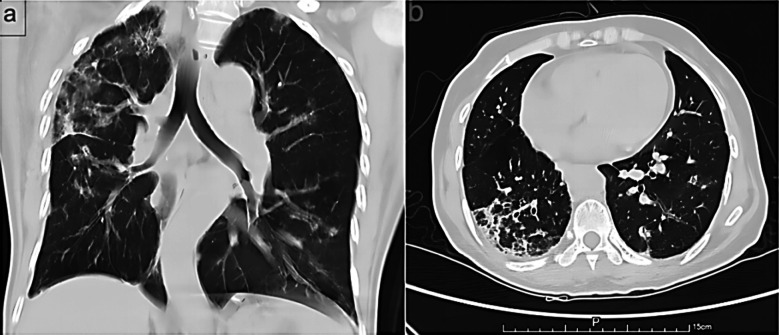
CT of the chest showed her bilateral pulmonary inflammatory infiltration, noted in the right (a, b).

A 65 years old female patient was admitted to our hospital complaining of limbs numbness for two days. Her numbness was predominantly on the right side and distal body parts, worsening when walking but slightly ameliorating during rest. Laboratory examinations results are showed in Table-I. Upon examination, it indicated that the patient experienced acute kidney injury (AKI) along with a positive ANCA. Specifically, the titers of myeloperoxidase (MPO), exhibited a significant increase. On admission, her physical examination revealed positive Romberg signs and decreased pain and temperature sensation in the dorsum of her right hand and below both ankle joints. Chest computed tomography (CT) scans showed her bilateral pulmonary infiltration of inflammation, noted in the right (Fig.1). Electromyography test for her showed polyneuropathy involving both upper and lower limb motor and sensory nerve axons, some with myelin sheath involvement. Brain magnetic resonance imaging showed her ischemic changes in white matter and age-related brain changes.

**Table-I:** Lab examination in our hospital

Variable	Normal Range	Day 1	Day 3	Day 7	Day 14	Day 21
WBC	4-10 (×10^9^/L)	12.79	10.51	10.09	15.04	14.6
Hb	110-150(g/L)	105	92	82	92	103
Lymphocyte	1.2-4.8(×10^9^/L)	0.96	1.17	1.02	1.46	2.39
Neutrophil	1.5-8.0(×10^9^/L)	10.41	8.48	8.41	12.71	11.32
Platelet	100-300(×10^9^/L)	451	394	405	399	355
Urine WBC	0-5.4(↑/HPF)	14.32	12.94	14.86	8.56	4.78
Urine RBC	0-4.5(↑/HPF)	98.75	108.77	78.41	34.76	22.13
Urine Protein	-	++	++	++	++	++
TP	65-85[g/L]	/	70.8	62.1	61.4	63.1
ALB	40-55[g/L]	/	28.5	23.3	28.5	30.8
24hUpros	0-150mg	/	2782	1614	1432	1078
Urea	2.60-7.50mmol/L	9.8	11.06	6.37	11.27	13
CREA	41.0-73.0umol/L	98	97.8	106.8	135	112.7
ANA	<1:100	/	>1:3200	/	/	1:1000
c-ANCA	-	/	+	/	-	-
p-ANCA	-	/	++	/	++	+
MPO-Ab	0-20(RU/mL)	/	142.45	/	105.3	86.4
PR-3	0-20(RU/mL)	/	<20		<20	<20
Anti-RNP	-	/	+++	/	++	+
IgA	1-4.2[g/L]	/	6.91	/	3.78	2.12
IgG	8.6-17.4[g/L]	/	18.2	/	15.9	14.3
IgM	0.3-2.2[g/L]	/	1.97	/	1.56	1.17
CRP	0-8(mmol/L)	/	83.8	79.7	30	5.86
C3	0.79-1.52[g/L]	/	1.26	/	1.45	1.06
C4	0.16-0.38[g/L]	/	0.23	/	0.21	0.19
ESR	0-15(mm/h)	/	117	122	70	51

**WBC:** White blood cell, **RBC:** Red blood cells, **Hb:** hemoglobin, **TP:** total bilirubin, **ALB:** albumin, **24hUpros:** 24 hours urine proteins, **CREA:** creatinine, **ANA:** anti-nuclear antibody, **c-ANCA:** clonal-anti neutrophil cytoplasmic antibodies, **p-ANCA:** polyclonal-anti neutrophil cytoplasmic antibodies, **MPO:** myeloperoxidase, **PR-3:** proteinase 3, Anti-R**NP:** anti-nuclear ribonucleoprotein antibody, **Ig:** immune globulin, **CRP:** C-reactive protein, **C3:** complement 3, **C4:** complement 4, ESR erythrocyte sedimentation rate.

The patient was diagnosed with AASV and AKI. Then, pulse therapy of methylprednisolone (500mg once daily, intravenous drip for three days), followed by oral administration (40mgonce daily) and cyclophosphamide (50mgtwice daily) were prescribed for her. Two weeks after, her numbness improved significantly. However, there was still a decrease in sensation at the fingertips and soles of both feet. Assisted acupuncture and physical treatment, the patient’s discomfort gradually alleviated. Follow-ups out-patient showed her a significant improvement in her overall condition and no evident adverse drug reactions have been observed.

## DISCUSSION

In the early stages of AASV, the surrounding neurological blood supply is rich and collateral circulation is sufficient, making it generally less susceptible to damage. However, as the disease progresses, approximately 70% of patients may experience neurological complications.^3^ The most common manifestation is multiple mononeuritis, which begins with involvement of a single nerve and gradually progresses to affect multiple nerves.

In addition to sensory impairment, motor dysfunction is often present, highlighting the characteristic feature of AASV affecting the peripheral nerves. This patient exhibited acute onset, with numbness in the limbs as the first symptom, primarily on the right side and distal parts. This is a typical clinical manifestation of PN, which is often caused by vasculitis in the vessels supplying the nerves, leading to ischemia in nerve tissues. The ischemic process inflicts damage to PN and axonal degeneration, resulting in motor and sensory dysfunction in these patients.^4^

In clinical practice, patients diagnosed with AASV should have specialized neurological consultation and examination. Treatment for AASV in this case involved pulse therapy of glucocorticoid (GC) and combined immunosuppressive therapy, which gradually alleviated the patient’s numbness and significantly improved her overall condition. GCs have long been considered first-line therapeutic agents for inducing remission in AASV. Combined treatment of cyclophosphamide and GC raises the remission rate to 93% and lowers the mortality and recurrence rates of patients with AASV.^5^ However, the recurrence rate of AASV is about 50% within five years, regardless of the medication used for maintenance treatment.^6^ Therefore, it is crucial to conduct regular follow-up and individualized treatment for patients with AASV.

Some studies in recent years have found that Rituximab, an antibody specially engineered to bind with CD20 positive B cells, has come to the forefront as a pivotal component in the therapeutic approach to AASV.^7^ This treatment introduces an innovative action mechanism that manipulates the very immune pathway implicated in AASV, thereby circumventing the need for broad-spectrum immunosuppression.^8^ Clinical trials have indicated promising outcomes with rituximab use, showcasing its effectiveness not solely as a preliminary induction treatment but also as a subsequent maintenance strategy. Research findings highlight significant reductions in relapse rates, particularly when juxtaposed against conventional cytotoxic substances or continued azathioprine therapy.^9^ Since it is not a first-line agent for AASV and the patient was effectively treated with glucocorticoids in combination with cyclophosphamide, rituximab was not used for the time being in this case.

### Limitations of the study

One of the principal limitations arises from the patient’s refusal to undergo a renal biopsy, leading to a lack of renal pathology evidence that could have been potentially significant. However, this does not compromise the integrity of our diagnostic and therapeutic approach for this patient. Furthermore, it must be acknowledged that the follow-up duration for this patient has been relatively short. As of now, there have been no notable adverse reactions. Looking ahead, the goal and dedication remain steadfast in continuing to monitor this patient during the subsequent phases, aiming to provide a more thorough and continuous evaluation of their health status.

## CONCLUSION

AASV with PN as the initial symptom remains rare. This case emphasizes the importance of including AASV in the differential diagnosis of unexplained neurological symptoms. Early diagnosis and appropriate treatment are crucial for improving the survival rate and functional prognosis of patients with AASV.

### Consent

The patient provided her written informed consent to participate in this study. Written informed consent was obtained from the individual for the publication of any potentially identifiable images or data included in this article.

### Authors’ contributions:

**XL:** Study design, manuscript revision and responsible for the content of the article and the accuracy of the data.

**XH:** Manuscript writing.

**YW:** Data and image analysis, literature review.

All authors contributed to the article and approved the submitted version.

## Data Availability

The original contributions presented in the study are included in the article, further inquiries can be directed to the corresponding author.
